# Health Professionals' Attitudes Towards Traditional Healing for Mental Illness: A Systematic Review

**DOI:** 10.1111/inm.70043

**Published:** 2025-04-21

**Authors:** Alemayehu Molla Wollie, Kim Usher, Kylie Rice, Md. Shahidul Islam

**Affiliations:** ^1^ School of Health, Faculty of Medicine and Health University of new England Armidale New South Wales Australia; ^2^ Department of Psychiatry, College of Medicine and Health Sciences Injibara University Injibara Ethiopia; ^3^ School of Psychology, Faculty of Medicine and Health University of New England Armidale New South Wales Australia

**Keywords:** attitudes, health professionals, mental illness, systematic review, traditional healing

## Abstract

Combining modern treatments with traditional healing approaches has been proposed as one way to address mental health problems, especially in low‐income countries where the costs of pharmaceuticals often prevent or reduce their use. Despite health professionals' involvement being crucial for the integration of this approach, their involvement has been limited to date. This systematic review is designed to explore the attitudes of health professionals towards traditional healing practices for mental illness. The Preferred Reporting Items for Systematic Review and Meta‐Analysis (PRISMA) 2020 guidelines were followed. The studies were identified from Scopus, EMBASE, PubMed, PsycINFO, and the Web of Sciences. The qualities of the included articles were assessed using the Mixed Method Appraisal Tool (MMAT) Version 2018, and mixed‐method synthesis was used to narrate the results. Of the 2115 identified articles, 36 were included in the data synthesis. From the extracted data, health professionals had negative, mixed, and positive views towards traditional healing approaches for mental illness. Their negative attitude towards traditional healing approaches were due to their concerns that traditional healing may cause harm to service users, and they have no trust in the scientific basis, education, or practices of healers. Despite the fact that it is crucial for healthcare professionals to comprehend the cultural backgrounds of those receiving mental health services in order to offer care appropriately, health professionals' negative and mixed attitudinal expressions towards traditional healing approaches limit their involvement. This might be improved by identifying barriers from the perspective of practitioners and creating culturally appropriate guidelines for communication and referral between traditional healing approaches and biomedical care.

## 1 Introduction

Mental illness is a significant public health problem and accounts for 32.4% of years lived with disability worldwide (Vigo et al. 2016). It is related to psychological, physical, social, cultural, and spiritual factors (Ashouri et al. 2016; Lamond et al. 2015) and has a substantial impact on a person's feelings, thoughts, behaviour, and social interactions (Armijo 2017; Marggraf et al. 2020). Mental health issues are a global health concern and are highly prevalent, particularly in low‐income countries (Esan and Esan 2016; Fisher et al. 2012). Lower‐income countries have fewer health professionals (Bruckner et al. 2011; Juengsiragulwit 2015), resulting in a treatment gap for people with mental illnesses (van den Broek et al. 2023). Negative attitudes, stigma, limited resources, and low priority are common reasons for the high burden of mental illness, in addition to a lack of health professionals in lower‐income countries (Corrigan et al. 2014; Jenkins et al. 2013; Monteiro 2015; Musyimi et al. 2018).

Traditional healers are people who practice methods such as faith or spiritual therapies, counselling, ceremonies, plant‐based remedies, and related treatment approaches (Nortje et al. 2016). Traditional healing is a body of knowledge and a set of beliefs that use culturally accepted spiritual treatments and plant products to identify and treat disease (Che et al. 2024). About 80% of Asians and Africans use traditional treatment for their healthcare (Oyebode et al. 2016), and traditional healers are frequently consulted by people with symptoms of mental illness in lower‐income countries (Cianconi et al. 2019; Herman et al. 2018; Nortje et al. 2016). Traditional treatments are more commonly practiced in lower‐income countries; for example, traditional healers are widely consulted in South Africa (Mohamed‐Kaloo and Laher 2014; Mokgobi 2014; van Rensburg et al. 2014), Ghana (Arias et al. 2016; Asafo 2021; Asamoah et al. 2014; Krah et al. 2018), Nigeria (Anjorin and Wada 2022; Jidong et al. 2021; Olutope 2020), and Ethiopia (Baheretibeb et al. 2024; Belachew et al. 2019; Gutema and Mengstie 2023). Traditional healers are called by ancestors to become healers or obtain their knowledge through intensive learning about traditional medicines and occupy themselves with multiple healing practices (Zuma et al. 2016). In addition, they are descended from branches of the extended family that have experience in counselling, interpreting the Bible, and employing prayer aids such as holy water and oil as treatment means (Baheretibeb et al. 2024; Kpobi and Swartz 2018). People choose traditional healing approaches due to affordability, perceived effectiveness, and community accessibility (Hailu et al. 2020; Ibrahim Awaad et al. 2020).

Though many people use traditional treatments, health professionals tend to differ in their opinions regarding traditional healing approaches (James et al. 2020). This is due to their philosophical differences, such as considering that traditional treatments are not scientific or evidence‐supported (Hamilton and Marietti 2017) and their notion that traditional healers do not treat people with mental health issues with compassion or from a human rights perspective (Bitta et al. 2019; Nyame et al. 2021). In another study, healers and health professionals have agreed on the benefits of collaborative treatment for service users, but they have disagreed on safety issues and disease conceptualisations (Green and Colucci 2020). Likewise, modern health professionals lacked trust and were unwilling to refer their patients to traditional healers (Lampiao et al. 2019).

According to another study, traditional healers can offer psychosocial support for mental health issues, particularly for common mental illnesses like depression and anxiety (Nortje et al. 2016). Furthermore, the acceptability and effectiveness of traditional treatment for mental illness were recently reported (Nwagbo and Moses 2022), and patients receiving traditional treatment for psychotic disorders showed some improvement after using traditional treatments (Sorketti et al. 2013). However, there is insufficient evidence to support the effectiveness, standardisation, and recommendation of traditional treatment as a first‐line treatment for schizophrenia (Ngubane et al. 2024; Ojagbemi and Gureje 2020).

To address the treatment gap for mental illness, particularly in resource‐limited countries, the World Health Organisation (WHO) has recommended governments incorporate evidence‐based traditional treatment approaches as treatment sources (WHO 2021). In addition, the Mental Health Gap Action Program (mhGAP) recognises the value of holistic care by engaging non‐specialists in mental health care (Keynejad et al. 2018). Likewise, the review study has also shown the consideration of traditional healers as resources for mental health in policies (Blignault and Kaur 2020). Despite the above attempts, attitudinal differences, reluctance to recognise non‐specialists' role in mental health care, and stigma towards mental health care limit its implementation (Badu et al. 2019; Masemola et al. 2023). To implement policies and guidelines on holistic care for mental health problems, health care providers involvement is crucial. In addition, in order to manage and counsel patients appropriately and empower them towards a good recovery process, health care providers should have a thorough understanding of the cultural beliefs and practices of their patients (Masemola et al. 2023), since service users need community‐oriented approaches to adhere to treatments. Improved communication and collaboration between health professionals and traditional healers are necessary to provide affordable care and bridge the gap in treatment for mental illness.

Thus, the aim of this systematic review was to summarise health professionals' attitudes towards traditional healing approaches for mental illness.

## 2 Methods

The systematic review was carried out using the Preferred Reporting Items for Systematic Review and Meta‐Analysis (PRISMA) 2020 guidelines (Page et al. 2021). In addition, we have used the PRISMA checklist to prepare the manuscript for this review. This review has been registered with the International Prospective Register of Systematic Reviews (PROSPERO) with a registration number CRD42014535136. The protocol is also published in PLoS One journal (Wollie et al. 2024).

### 2.1 Inclusion Criteria

All published studies that focused on outcome variables and conducted among health professionals were included in this review. The outcome variables for this study were attitudes, which were expressed as negative (Lampiao et al. 2019; Musyimi et al. 2016; Shields et al. 2016), moderate or mixed (Liem 2020; Tehrani et al. 2023), and positive (Ho et al. 2016; Liem and Newcombe 2021) in primary studies. In addition, studies that focused on traditional healings for mental illness (Nortje et al. 2016) were considered in this review. Likewise, all published qualitative, quantitative, and mixed studies were included without restrictions in the study designs. Moreover, articles written and published in English from January 2014 to April 2024 were included to summarise contemporary data on the topic.

### 2.2 Exclusion Criteria

Studies that didn't incorporate health professionals and didn't include mental illness were excluded from this review. In addition, studies that did not focus on the outcome variable (attitude) toward traditional healing were excluded from the study. Furthermore, non‐English publications, publications before 2014, conference summaries, dissertations, governmental and non‐government reports, and letters were excluded from the study.

### 2.3 Search Strategy

A literature search of databases such as Scopus, PsycINFO, PubMed/Medline, EMBASE, and the Web of Sciences was conducted. The key terms were searched, connecting Boolean operators AND/OR to address articles. Searching strings for databases were (attitude OR perception OR belief OR opinion OR view) AND (“health professionals” OR “health practitioners” OR “health personnel” OR nurses OR “medical doctors” OR psychiatrist OR psychologist) AND (“traditional healing” OR “traditional medicine” OR “herbal medicine” OR “indigenous treatment” OR “spiritual therapy” OR “religious healing”) AND (“mental illness” OR “mental disorder” OR “mental health” OR “psychological distress” OR “psychiatric disorder”). The reference lists of the included articles are screened to address the remaining studies. The selected databases, search terms, and initial screening processes were finalised in consultation with a senior health librarian from the University of New England (UNE) Dixon Library and the authors listed in this paper.

### 2.4 Study Selection Process

The selection process began by importing all outputs into the EndNote library. Duplicates were removed before starting the screening. The first author conducted the initial screening, and this was verified by another author. The reviewers evaluated article titles and abstracts, retaining those that were important to the outcome variable (attitude) of health professionals towards traditional healing for mental illness. Finally, the full texts of possible relevant papers were evaluated for eligibility using predetermined inclusion and exclusion criteria.

### 2.5 Data Extraction

In the beginning, the first author extracted data, which was confirmed by the second author and the research team. The main findings were extracted from the abstract part, main body result, and conclusion sections. For quantitative studies, summarised percentages and mean scores on attitudes were used for data extraction. For qualitative studies, self‐explicit quotes of participants within the main result body and summarised text reports from the result and the conclusion parts of the abstract were extracted. The first author's name, the year of publication, the study approach used, the health professional type, the data collection and analysis method, the main findings, the country in which the study was carried out, and the conclusions were used in the data extraction template.

### 2.6 Quality Assessment

The quality of each study was evaluated in order to ascertain the validity of the findings. The Mixed Methods Appraisal Tool (MMAT) Version 2018 Checklist (Hong et al. 2018) was used to assess the qualities of the included articles. This tool is designed to assess the methodological qualities of different study designs (i.e., quantitative, qualitative, mixed‐method), with two common required questions and five distinct questions, for a total of seven questions for each. Based on a prior study that used a similar methodology, the articles were categorised with a score below 4 as low quality (LQ), 4–5 as medium quality (MQ), and 6–7 as high quality (HQ) (Fatema et al. 2021). The differences in quality assessment were resolved by team discussion.

### 2.7 Data Synthesis

After independent extraction of qualitative and quantitative data, the results of the quantitative studies were converted to qualitative results based on previously conducted studies and Joanna Briggs Institute guidelines (Aromataris et al. 2024; Kasse et al. 2024; Popay et al. 2017; Stern et al. 2021). Next, qualitative and quantitative data were integrated into a single synthesis using an integrated mixed‐method synthesis procedure (Sandelowski et al. 2006). Then, we read the extracted data frequently to become familiar with basic concepts. After reading the data multiple times, we revised and organised the data to establish groups based on concepts that were repeatedly mentioned. Finally, the categories were validated based on conceptual similarities to the outcome variable, and reporting was carried out using narrative synthesis.

## 3 Results

**FIGURE 1:**
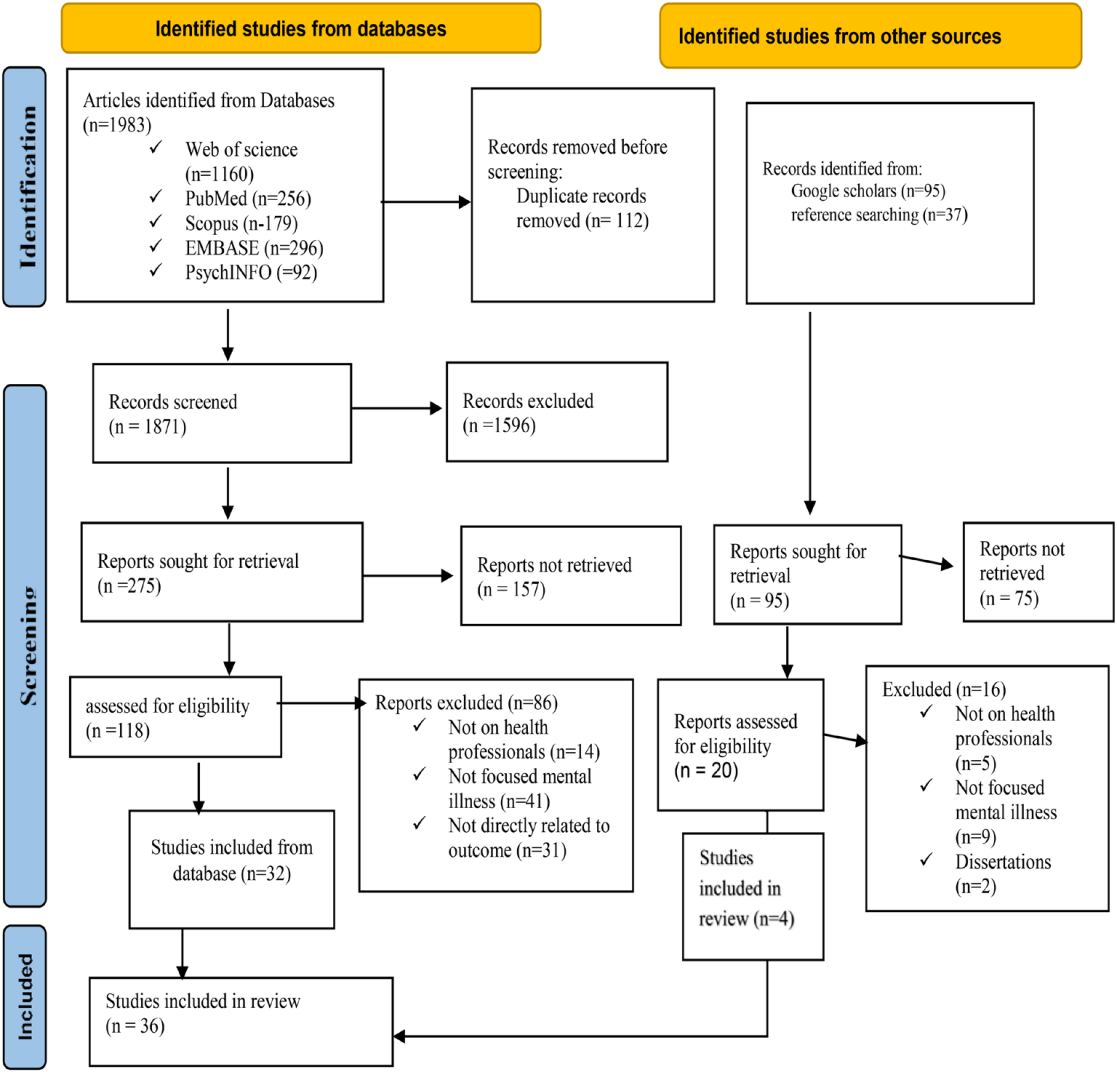
Study selection flow diagram (a model of PRISMA 2020).

From the total of 2115 initially identified articles, 1983 were obtained from electronic databases: Web of the Sciences (*n* = 1160), EMBASE (*n* = 296), PubMed (*n* = 256), PsycINFO (*n* = 92), and Scopus (*n* = 179). In addition, 132 articles were identified from Google Scholar search and reference lists of selected articles. From the identified articles, duplicates (*n* = 112) were automatically removed before screening. After screening abstracts and titles, 118 papers were evaluated based on eligibility criteria. Finally, 36 articles were included in the final data analysis (Figure 1).

### 3.1 Characteristics of Included Articles

Nearly half of the articles were conducted in African countries; five studies were from South Africa (Masemola et al. 2023; Mohamed‐Kaloo and Laher 2014; Mokgobi 2014; van Niekerk et al. 2014; van Rensburg et al. 2014). Of the total included articles, three were conducted in the United States of America (Earl et al. 2022; Lawrence et al. 2014; Neathery et al. 2020), Ghana (Arias et al. 2016; Kpobi et al. 2024; Nyame et al. 2021), and India (Khosla and Goel 2021; Ramakrishnan, Dias, et al. 2014; Shields et al. 2016) each. Two were from Australia (Hamilton and Marietti 2017; Thomson‐Casey et al. 2024), China (Ho et al. 2016; Ye et al. 2020), Malawi (Kokota et al. 2022; Lampiao et al. 2019), Tanzania (Patterson 2023; Solera‐Deuchar et al. 2020), Kenya (Bitta et al. 2019; Musyimi et al. 2016), and Indonesia (Liem 2020; Liem and Newcombe 2019) each. One article was conducted in three countries (Ghana, Kenya, and Nigeria) (Van der Watt et al. 2017). In addition, one study was conducted in South Africa and Rwanda (Schierenbeck et al. 2018), a single article each from Iran (Tehrani et al. 2023), Pakistan (Khan et al. 2023), Nepal (Pham et al. 2021), the United Kingdom (Sharp et al. 2018), and Croatia (Kralj and Kardum 2020). Regarding the study approaches, 24 included qualitative study methods, 11 employed quantitative, and one mixed methods study. A large number of the qualitative studies used interviews for data collection, and the majority used thematic analysis for their data analyses; four used ground theory approaches. The majority of study participants were mental health care professionals (Table 1).

**TABLE 1:** The characteristics of included articles in the systematic review.

Authors	Country	Aim of the study	Study Participants	Approaches	Sample size	Data collection methods	Data analysis method	Quality score/5
Arias et al. (2016)	Ghana	To examine the beliefs and practices of prayer camp staff and biomedical care providers perspectives	Nurse and General practitioners	Qualitative	31	Interview	Constant comparative/Ground theory	High
Bartholomew and Gentz (2019)	Namibia	To understand how mental health practitioners in Northern Namibia view their work with Aawambo Namibians	Mental health practitioners	Qualitative	7	Interview	Grounded theory	High
Bitta et al. (2019)	Kenya	To explore the local terms, perceived causes, and modalities of management for priority mental, neurologic, and substance disorders listed in the mhGAP	Primary health care providers	Qualitative	34	Focus group discussion		High
Earl et al. (2022)	USA	To understand mental health professionals' attitudes about what shaped provider and patient motivation to engage in religious and spiritual discussions	Health care providers	Qualitative	38	Focus group discussion	Thematic	High
Hamilton and Marietti (2017)	Australia	To explore the knowledge, attitude, and experience among psychologists towards complementary and alternative medicine	Psychologists	Qualitative	18	Interview	Thematic	
Hassan and Omer (2020)	Sudan	To explore the attitudes of psychiatrists and psychiatry trainees towards traditional healers in Sudan	Psychiatrist and psychiatry trainee	Quantitative	108	Self‐Administered	SPSS	High
Ho et al. (2016)	China	To investigate the roles and meanings of spirituality from the perspective's mental health professionals and people with schizophrenia	Mental health professionals	Qualitative	19	Interview	Grounded theory	High
Jidong et al. (2021)	Nigeria	To explore how traditions and cultural beliefs are integral to understanding indigenous mental health conditions and traditional healing.	Mental health care practitioners	Qualitative	26	Interview	Thematic	High
Khan et al. (2023)	Pakistan	To explore perceptions about treatment methods provided by different practitioners, as the identification of avenues for support can improve psychiatric, alternative interventions, and social outcomes	Health care providers	Qualitative	5	In‐depth Interview	Thematic	High
Khosla and Goel (2021)	India	To compare attitudes and opinions about traditional healing approaches with those biomedical techniques	Medical practitioners	Qualitative	2	In‐depth Interview	Thematic content analysis	High
Kokota et al. (2022)	Malawi	To investigate the views and experiences of western and traditional practitioners on potential combinations in the care of people living with mental illness	Nurses, medical assistants, clinical officers	Qualitative	Not mentioned	Key informant Interview and focus group discussion	Thematic	High
Kpobi et al. (2024)	Ghana	To develop collaborations between healers and formal health services to optimise available mental health interventions in poorly resourced contexts	Community mental health Providers	Qualitative	8	Visual ethnography and filmed individual interviews	Thematic	High
Kralj and Kardum (2020)	Croatia/Southeast Europe	To examine the variation in attitudes and beliefs towards alternative and complementary medicine, beliefs in afterlife and religiosity	Psychiatrist, Psychologist, and theologian	Quantitative	107	Self‐Administered	Descriptive analysis by R‐Software	High
Lampiao et al. (2019)	Malawi	To evaluate the challenges that exist between traditional healers and biomedical practitioners	Health care providers	Qualitative	11	Interview	Manual thematic analysis	High
Lawrence et al. (2014)	USA	To measure primary care physicians' and psychiatrists' awareness of religious mental health‐care providers and their willingness to refer there	Primary care physicians and Psychiatrists	Quantitative	1208	Self‐administered Via email	Saturated analysis/Post hoc analysis	High
Liem and Newcombe (2019)	Indonesia	To explore clinical psychologists' knowledge, attitudes, and usage of complementary‐alternative medicines in Indonesia	Clinical Psychologist	Quantitative	274	Self‐administered Via email	Descriptive/SPSS	High
Liem (2020)	Indonesia	To investigate the views about and experiences with religion and spirituality held by Indonesian clinical psychologists	Clinical Psychologist	Qualitative	43	Interview	Thematic	High
Masemola et al. (2023)	South Africa	To assess how mental health professionals feel about traditional medicine being integrated into the mental healthcare system	Psychiatrists and Psychiatric Nurses	Quantitative	85	Self‐administered	Descriptive EPI‐Info	High
Mohamed‐Kaloo and Laher (2014)	South Africa	To investigate Muslim general practitioners' views about mental illness	Health Practitioners	Qualitative	10	Interview	Thematic content analysis	High
Mokgobi (2014)	South Africa	To assess South African‐based western‐trained health professionals' attitudes towards traditional African treatment	Health care providers	Quantitative	319	Self‐administered	Non‐parametric Kruskal‐Wallis Test	High
Musyimi et al. (2016)	Kenya	To establish integration among informal and formal practitioners to improve community‐based mental health	Registered nurse and clinical officers	Qualitative	8	Focus Group discussion	Thematic content analysis and SPSS	High
Neathery et al. (2020)	USA	To investigate perceived barriers to providing spiritual care and how these were associated with spiritual care among nurses	Psychiatry Nurses	Quantitative	159	Online/Self‐Administered	Descriptive correlation analysis	High
Nyame et al. (2021)	Ghana	To explore the possibility of forging partnerships at the primary health care level in geopolitical regions in Ghana	Community mental health Providers	Qualitative	23	Focus group discussion	Thematic	High
Patterson (2023)	Tanzania	To examine the ways power and struggle for public authority impacts mental health services	Health care providers	Qualitative	33	Interview	Narrative	High
Pham et al. (2021)	Nepal	To explore how the public, traditional healers, and biomedical professionals view the different methods of services and make decisions regarding using either or both types	Physicians	Qualitative	5	In‐depth In review	Grounded theory practices	High
Ramakrishnan, Dias, et al. (2014)	India	To examine the perspectives of Indian traditional and complementary medicine and allopathic professionals on the influence of religion and spirituality on health	Mental health professionals	Quantitative	201	Self‐administered	Logistic regression	Medium
Schierenbeck et al. (2018)	Rwanda &South Africa	To explore mental health care professionals' responsibility for mental health care implementation and its relation to the intersection between modern and traditional medicine	Health care providers	Qualitative	20	Interview	Thematic	High
Sharp et al. (2018)	UK	To explore the views and experiences of professionals towards CAM for comorbid patients and the potential for integration into primary care	Health care providers	Qualitative	20	Focus group discussion	Content analysis	High
Shields et al. (2016)	India	To examine the use, origins, and outcomes of a combined program between allopathic and faith‐based care	Mental health practitioner	Qualitative	3	Interview	Thematic	High
Solera‐Deuchar et al. (2020)	Tanzania	To explore the views of biomedical and traditional providers towards collaboration between the two sectors	Nurse	Qualitative	6	Focus group discussion	Thematic	High
Tehrani et al. (2023)	Iran	To evaluate the use and perception of complementary medicine for mental illness among Iranian psychologists	Psychologists	Quantitative	100	Census	Descriptive analysis	High
Thomson‐Casey et al. (2024)	Australia	To determine the types Complementary medicine products, practices, and practitioners who are recommended and/or referred by Australian psychologists as part of their clinical practice, as well as investigate the relationship between psychologists' perspectives on the risk and importance of engaging wit complementary medicine in psychology	Psychologist	Quantitative	201	Self‐Administered via email	SPSS	High
Van der Watt et al. (2017)	Ghana, Kenya, Nigeria	To explore the scope of integrative treatment for persons with mental disorder as implemented by health professional, faith healers, and traditional healers	Health Care providers	Qualitative	57	Focus group discussion	Thematic	High
van Niekerk et al. (2014)	South Africa	To identify traditional healers' knowledge of occupational therapy, their use in interventions, and the allopathic health providers' perception of traditional practitioners' role in treating patients with mental health issues	Health care providers	Mixed/pilot study	34	Self‐Administered	Descriptive	Medium
van Rensburg et al. (2014)	South Africa	To examine the views of local psychiatrists on collaboration and referral between psychiatrists and religious or spiritual care providers	Psychiatrist	Qualitative	13	In‐depth Interview	Content analysis	High
Ye et al. (2020)	China	To examine the attitudes and practice of traditional and complementary medicine among leading psychiatrists, and to explore the possibility of future cooperation in China	Psychiatrist	Quantitative	49	Self‐administered	SPSS	High

### 3.2 Quality Assessment of Included Articles

The qualities of the included studies were evaluated using the Mixed Methods Appraisal Tool (MMAT) Version 2018 Checklist (Hong et al. 2018). All (36) of the included articles were eligible for the two common questions of the mixed‐method quality appraisal tool. Despite MMAT having no standard of scoring, based on a previously conducted study's scoring range (Fatema et al. 2021), 32 qualified for each of the five criteria, two qualified for the four criteria, and the remaining two qualified for the three assessment criteria. For the articles eligible in four criteria, sample size issues (Khosla and Goel 2021) and low response rates were reasons that reduced their scores (Lawrence et al. 2014). Among articles that fulfilled three criteria, the response rate was very low (49%) (Ramakrishnan, Dias, et al. 2014), and there is no clear integration of quantitative and qualitative results in one mixed‐method study in South Africa (van Niekerk et al. 2014). Although two studies scored low in separate five‐items quality assessment criteria, their average scores according to the total seven‐item criteria were within the medium quality range. As a result, we didn't exclude articles due to their quality criteria.

### 3.3 Result Synthesis of Included Studies

In accordance with the observation of the extracted data, negative, mixed, and positive attitudes were identified as categories among health professionals towards the traditional healing approach for mental illness. Participants with negative attitudinal expressions often reported concerns about power dynamics or role dynamics, as well as a lack of trust in the effectiveness and safety of traditional treatments. Among health professionals with mixed attitudes, referral systems and traditional treatment safety concerns were also cited as fundamental areas of ambiguity. However, participants who positively rated traditional treatment for mental illness acknowledged the practice of treatment in communities and its usefulness in some mental health problems (Table 2).

**TABLE 2:** Identified themes for the attitudinal expressions of health professionals towards traditional healing for mental illness.

Major themes	Sub‐themes
Negative attitudes	Related to role superiority or power dynamics issues
	Related to effectiveness issues
	Related to the safety of traditional healing approaches
Mixed attitudes	Related to safety
	Related to referral
Positive attitudes	Largely recognised and practiced within the community
	Perceived effectiveness of traditional healing for mental illness

### 3.4 Negative Attitudes Towards Traditional Healing

The 14 studies showed that health professionals were not supportive of traditional approaches for mental illness. Their positions were commonly related to their feelings of superiority and their concerns about effectiveness and safety issues with traditional healing approaches.

#### 3.4.1 Role Superiority or Power Dynamic Issues

The superiority was largely mentioned as an issue related to the negative expression of health professionals towards traditional healing for mental illness. The study conducted in India showed that mental health care practitioners reported difficulty in collaboration due to perceived differences in professional and social status between the two systems, which was explained as “not possible because doctors' and faith practitioners' statuses are different” (Shields et al. 2016, 374). In another study, general health care practitioners were the least enthusiastic and saw complementary and alternative medicine providers' roles as limited in treating mental illnesses (Sharp et al. 2018). The study conducted in Kenya, Nigeria, and Ghana noted that health care providers showed distrust and a sense of superiority, and some of them held the perception that traditional healing may be an obstacle to their profession. For example, “They should be removed from the system; we may actually accept this if they can give us the evidence base of their intervention, how they work, and when they should work” (Van der Watt et al. 2017, 2/80). In another study conducted in the USA, health care providers reported misconceptions about the roles and responsibilities of spiritual healers (Earl et al. 2022). Similarly, health professionals lacked clarity on the benefits and role of traditional healing, such as “It is not clear how patients benefit from traditional healers” (Musyimi et al. 2016). “There is no common consensus on what role each entity plays, especially the traditional and faith healers.” “Sometimes we feel that the patient would not have confidence in us if we referred patients to traditional or faith healers” (Musyimi et al. 2016, 5). The study conducted in South Africa showed that health care providers were not willing to recognise the roles of traditional healers in providing health care services (van Niekerk et al. 2014).

#### 3.4.2 Effectiveness Issues of Traditional Treatments

The study conducted in Malawi found that health professionals are not willing to refer their patients to traditional practitioners due to several reservations, like lack of trust due to the absence of a scientific basis, education, and an inability to understand medical evidence among traditional healers (Lampiao et al. 2019). Similarly, psychiatrists in South Africa noted that referring patients to spiritual healers is challenging, and integrating them into the existing mental treatment team is very difficult (van Rensburg et al. 2014). In another study, most of the clinicians focused on the psychosocial and biologically related causes of mental illness, and they reported that patients' first contact with traditional healers was a major barrier to treatment effectiveness (Khan et al. 2023).

#### 3.4.3 The Safety of Traditional Healing Approaches

The study conducted in Ghana indicated a negative attitudinal expression of health professionals towards traditional healers; they believe traditional healing may cause harm to service users, and even this may be human rights abuse. As a result, they insisted that any form of partnership would first require an investigation of traditional practices, like herbal preparation, which could be medically harmful to service users (Nyame et al. 2021). Primary health care providers were unwilling to collaborate with traditional health practitioners because they perceived traditional healing approaches as contributing to the worsening of patients' prognoses, according to a study conducted in Kenya (Bitta et al. 2019). A study conducted among physicians in Nepal showed very limited communication between traditional healers and modern physicians, and most of them suggested that formal referral should be one‐way only, that is, from traditional healers to physicians, and recommended traditional healers learn biomedical approaches rather than referring their patients (Pham et al. 2021). In another study, psychiatric nurses showed negative thinking, a lack of education about religious healing (Neathery et al. 2020, 575), and a fear of exacerbating symptoms if they considered traditional healing (Bitta et al. 2019; Neathery et al. 2020).

### 3.5 Mixed Attitudes Towards Traditional Healing

Some of the studies reported mixed attitudes of health professionals towards traditional healing for mental illness. According to the extracted data, their attitudinal expressions were more commonly related to the referral and safety issues of traditional healing approaches.

#### 3.5.1 Attitude Related to Safety

The study conducted in Australia noted that a small number of psychologists believe complementary medicine is not safe and puts people at risk (Thomson‐Casey et al. 2024). However, more than half of the participants were willing to recommend complementary or traditional healing, and one‐quarter of psychologists referred their patients when they perceived risks related to complementary medicine (Thomson‐Casey et al. 2024). While some psychologists have positive expressions towards alternative and complementary medicine for mental illness, there are also ambivalences and oppositions from some clinicians, according to another study conducted in Australia (Hamilton and Marietti 2017). The attitudinal ambivalence was due to psychologists adhering to scientific and patient‐centred approaches (Hamilton and Marietti 2017). In another study, practitioners expressed a moderate attitude towards traditional healing, and nearly half of them believed that the traditional approach could effectively treat psychiatric problems like schizophrenia (Mokgobi 2014). The study conducted in Indonesia showed mixed attitudes among participants towards spiritual therapy, particularly due to its effectiveness and safety issues (Liem 2020).

#### 3.5.2 Attitude Related to Referral

The study conducted in Sudan noted that nearly half of professionals are willing to discuss and support collaboration with traditional healers, but three‐quarters of them do not refer their patients to traditional healers (Hassan and Omer 2020). Similarly, the study conducted in Rwanda and South Africa showed varying views of health care providers; some believe collaboration between biomedicine and traditional healing is a possible means to prove cross‐cultural knowledge sharing among staff. Some respondents identified doubts about gains from working more closely with traditional healers in South Africa. In Rwanda, health professionals who participated were not inclined towards traditional healing approaches (Schierenbeck et al. 2018). Some biomedical care providers expressed positive opinions, but others insisted on identifying and giving biomedical treatment before referring or considering traditional healing, according to a study conducted in Ghana, where they were found to be against fasting activities practised in prayer camps as a means of treating mental illness (Arias et al. 2016).

### 3.6 Positive Attitudes Towards Traditional Healing

Whilst there was no article that identified only positive attitudes of health professionals among the included studies, some studies describe some positive expressions of health care professionals towards traditional healing for mental illness (Jidong et al. 2021; Kpobi et al. 2024; Mohamed‐Kaloo and Laher 2014). According to the original articles, their positive expression originated due to the recognition of traditional practice within the community and the perceived effectiveness of traditional healing for mental illness.

#### 3.6.1 Largely Recognised and Practiced Within the Community

The study conducted in Nigeria showed that a large number of medical practitioners have a positive attitudinal expression towards traditional healing, and they believe that traditional practitioners are valued figures in local communities and are widely consulted by service users (Jidong et al. 2021). In this study, traditional healers were recognised as readily available for patients (Jidong et al. 2021). Health professionals were aware that traditional healers and their followers also believed in spiritual power, recited biblical texts, and prayed with patients in a study conducted in Tanzania (Patterson 2023). As a result of their positive view, health care providers identified regularly consulted traditional healers for their patients, according to a study conducted in South Africa (Mohamed‐Kaloo and Laher 2014). Similarly, community mental health professionals in a study conducted in Ghana recognised and acknowledged traditional healing, and they emphasised that some professional training in mental health could give healers better insight (Kpobi et al. 2024).

#### 3.6.2 Effectiveness of Traditional Healing in Improving Symptoms of Mental Illness

The study conducted in Nigeria indicated that some symptoms are only improved by traditional healers (Jidong et al. 2021). Another study also showed the positive expression of professionals towards spirituality and identified regard for spirituality as a means of receiving support and managing symptoms (Ho et al. 2016). Psychiatrists witnessed how prayer and help from a religious community could improve patients, according to a study conducted in Tanzania (Patterson 2023). Another study conducted in Tanzania showed that most of the nurses accepted the traditional healer's role in mental illness treatment (Solera‐Deuchar et al. 2020). A study conducted in Malawi showed that, despite having no prior experience working together, many health care professionals have positive attitudes (Kokota et al. 2022), and approximately two‐thirds of primary care physicians and half of the psychiatrists have a positive attitude towards religious healing, and they refer their depressed or anxious patients to these religiously based practitioners in a study conducted in the USA (Lawrence et al. 2014). Similar to this, a study conducted in China showed the positive attitudes of leading psychiatrists and their experiences with the prescription and recommendation of traditional medicine for mental illness (Ye et al. 2020) (Table 3).

**TABLE 3:** Major findings and conclusions of included studies.

Authors	Main findings	Conclusion/Recommendations
Arias et al. (2016)	Although biomedical care providers expressed positive opinions towards integrative work, they noted the first‐time identification of biomedical problems and the administration of modern treatment. They also expressed their concerns about clients being encouraged to fast as traditional healing treatment means since lack of water and food could worsen situations and lead to bad health outcomes, and they identified chains as one of the most serious technique of prayer	Health professionals are interested in discussing with prayers
Bartholomew and Gentz (2019)	Three participants expressed traditional healing negatively, and they believe traditional healers work as fraudulent practices that disturb community and modern health, but others believe that there are genuine traditional healers who are effective. Some reported a clear difficulty and potential scepticism in integrating traditional practices and Western‐informed mental health services in Namibia, but four participants advocated for integrating their work with traditional healers	Health providers should further pursue avenues for integration and the role of cultural competence
Bitta et al. (2019)	Primary health care providers were unwilling to collaborate with traditional health practitioners because they perceived traditional healing approaches as contributing to the worsening of patient's prognoses	Case detection and referral may be hindered by lack of collaboration between traditional and primary health care providers
Earl et al. (2022)	Experiences and attitude towards religious and spiritual care were different. Certain medical workers were apathetic, only participating in conservation at the patient's requests. They have misconceptions about the roles and functions of spiritual healers. One participant feels that talking about spiritual therapy with patient is not necessary	Physicians and patients may develop more trust and confidence in advantages of chaplaincy service by making chaplaincy visible and approachable in outpatient settings
Hamilton and Marietti (2017)	While psychologists reported a positive attitude towards complementary and alternative approaches, there are also ambivalences and oppositions. The ambivalence was evident in the description of psychologists adhering to the scientific model and embracing a patient‐centered way, which affected their beliefs	Psychologists seem to benefit from various forms of complementary and alternative medicine, but they face unique challenges, like as ethical issues
Hassan and Omer (2020)	Nearly half of professionals are willing to discuss and support collaboration with traditional healers, but three quarters of them do not refer their patients to traditional healers. Most of them agree that traditional healers may have a role in the treatment of anxiety but not in the treatment of psychotic, mood, and personality disorders	Half of the participants in Khartoum supported collaboration with traditional healers
Ho et al. (2016)	Professionals have positive attitudes towards spirituality since they regard it as a means of receiving support and managing symptoms	The two perspectives' different understanding offer insight and even a roadmap for developing spiritual evaluation and holistic care
Jidong et al. (2021)	Almost all medical practitioners have a positive attitude towards traditional healing, and they believe that traditional healers are figures in communities and largely consulted by potential service users. As a result, most people do not see the clinician as their first point of contact when they have symptoms of mental illness. Traditional healers are easily available for patients, and there are also symptoms that are only treated by traditional healers	Traditional healing services have been in use across generations and are believed culturally acceptable and helpful
Khan et al. (2023)	Health professionals concentrated on the psychosocial and biological cause of mental illness. As a result, they have a negative attitude towards traditional healing, and this is explained by the fact that patients first visit to traditional healers is a major barrier to their overall treatment effectiveness. They come to us after knocking on lots of doors. Additionally, a clinician said that faith healers occasionally give false information about psychosis	The improving the treatment gaps and collaboration of traditional and clinical treatment is underscored
Khosla and Goel (2021)	One participant negatively expressed traditional healing practices, but the second participant has a good view of traditional services	The research findings are good evidences for developing a combination of traditional healing methods with biomedical treatment
Kokota et al. (2022)	They have no prior experience with collaboration; many have a positive attitude, but there are some HWs who have a negative attitude (I have never seen someone come in the open and testify that they have been healed after receiving medicine from TH)	With right procedures and respectful communication, integration between two is possible
Kpobi et al. (2024)	Health professionals have a positive attitudinal expression towards traditional healing; they recognise and acknowledge it, and they emphasise that professional training in mental health could give healers better insight	Institutional and logistical supports are recommended to ensure successful integration between healers and mental health workers
Kralj and Kardum (2020)	Psychologists and psychiatrist compared to theologians, have lower values regarding the relevance of faith in everyday life. Despite the average score being positive, there is no significant association, and as a result, it is possible to report that they have a negative attitude	The training of mental health practitioners should improve and enhance awareness of religion and spirituality. A qualitative study is also recommended for a deeper understanding
Lampiao et al. (2019)	Biomedical practitioners had a negative attitude; they luck trust on healers and they refused send patients to them due to several reservations, such, education, lack of absences of scientific base, and inability to understand medical teaching among traditional healers	While traditional healers demonstrated trust in biomedical practitioners, reciprocal trust from biomedical practitioners was lacking
Lawrence et al. (2014)	Approximately two‐thirds of primary care physicians and half of psychiatrists have a positive attitude towards religious healing, and as a result, more than half of them refer their depressed or anxious patients to these religiously based practitioners	Many physicians were willing to link patients to religious mental health providers
Liem and Newcombe (2019)	The majority (85.8%) have a positive attitude towards alternative and complementary medicine, and nearly half of them have personal experiences of using it	Participants reported low knowledge but positive attitudes towards CAM, especially integrating CAM into their clinical practice
Liem (2020)	Participants showed mixed attitudes towards spiritual therapy, particularly regarding its effectiveness. They have positive attitudes towards religious therapy. Generally, practitioners genuinely accepted that they were not able to detach spiritual and religious practices from their profession completely, and were willing to welcome collaboration	Professional organisations and psychological institutions should design and education and regulation of spirituality
Masemola et al. (2023)	Majorities of mental health professionals showed negative attitudinal expression towards traditional healing practices; they have concerns about the importance of traditional healing. The concern is based on the two treatment approaches being isolated from each other	There is doubt among healthcare providers on whether to support the collaboration of traditional medicine into their system or not
Mohamed‐Kaloo and Laher (2014)	General health practitioners have positive attitudes towards spiritual and traditional healing approaches for mental illness, and they have experienced regularly consulting traditional healers for their patients	Healthcare professionals understand the cultural and religious classifications sickness and how traditional healing is used as a mode of treatment
Mokgobi (2014)	Health care practitioners expressed moderate opinions about traditional healing. Psychiatric professionals' opinions were more favourable than those of nurses' general physicians. Over half of them viewed that traditional methods could effectively improve psychiatric problems such as schizophrenia	Psychiatric nurses and psychiatrists would be more willing to work with traditional healers than physicians and general nurses
Musyimi et al. (2016)	Health care providers have a negative view of traditional healing, as explained by their sayings in FGD. “It is not clear how patients benefit from traditional healers.” There is no common consensus on what role each entity plays, especially the traditional and faith healers. “Sometimes we feel that the patient would not have confidence in us if we referred patients to traditional or faith healers”	Information regarding how to start integration between the formal and informal sectors is lacking.
Neathery et al. (2020)	Mental health nurses reported that negative religious thinking and a lack of educational preparation are the most frequent barriers to spiritual care. They also have their internal feelings, like, “how can I support something I do not believe in?” As a result, they did not provide spiritual care more frequently. Majorities of psychiatric mental health nurses reported barriers to provide spiritual care, and they also fear exacerbating symptoms if they consider traditional healing	About 86% respondents in this study never received spiritual care education. Education about providing spiritual care is recommended
Nyame et al. (2021)	Negative attitudes towards traditional healers; they believe that THPs delay modern health care pathways, may cause harms to service users, and even this may be human rights abuse. Despite some acceptance of collaboration, they insisted that any form of partnership would first require an investigation of traditional practices, like herbal preparation, which could be medically harmful to service users	PHC providers need to be educated on accepting service users and their decision to seek treatment; this could include workshops on clarifying the value of relationships
Patterson (2023)	Health professionals were aware of traditional healers since some of their followers also believed in spiritual power; they recited biblical texts and prayed with patients as they dispensed medications, and even some organised workshops with traditional healers. A few cultivated informal ties with healers in order to train them about the signs of mental health crises. Psychiatrists also witnessed how prayer and help from a religious community could improve patients improve	Collaboration across approaches is recommended to inform knowledge
Pham et al. (2021)	Collaborative efforts are few in number, which indicates a negative attitude towards traditional healing, and one psychiatrist permitted his patient for conversion disorder, for only one‐time treatments from a healer. But formal referral is one way from traditional healers to physicians. Physicians recommended that traditional healers should learn biomedical approaches	Mutual understanding between both are recommended as the most viable options for collaborative work
Ramakrishnan, Dias, et al. (2014)	About three fourth of health care providers believe that spiritual therapy is beneficial and complementary to modern treatment for mental illness; large numbers of them reported feeling comfortable in discussing religious and spiritual concerns if the patient brings them up. They also believe that integrating both is good for service users	Both traditional and allopathic professionals are open to spirituality as a scientific academic subject
Schierenbeck et al. (2018)	Although some participants view collaboration between traditional medicine and biomedical care as a means to improve awareness between groups, there are participants who are sceptical of any benefit from doing together with traditional healers in South Africa. But in Rwanda, health professionals are not inclined towards traditional healing approaches	Policies and systems pertaining to health should recognise the importance of traditional medicine
Sharp et al. (2018)	General health care practitioners consider complementary medicine's role as limited in treating mental illnesses and comorbid MS problems. Despite integration may offer useful solution, some general practitioners have expressed worries that it could not be possible and would provide difficulties when incorporating complementary in to primary care	Collaboration alternative and complementary treatments into national health services is limited due to systematic challenges and ideological variation
Shields et al. (2016)	Mental health care practitioners showed a negative attitude towards collaborative work with traditional healing because of to their perceived disparities in societal and professional status. Initially, practitioners said, “This is not possible because doctors and faith practitioner's statuses are different”	Open discussion is important to reduce suspiciousness, increase knowledge exchange, and may be good for collaborative treatment approaches
Solera‐Deuchar et al. (2020)	Most of the nurses recognised the traditional practitioner's role in managing mental health issues, understand that many people with illness first contact healers, but one nurse believed that traditional practitioners' role was not relevant. They are willing to collaborate, but they expressed feeling unaware of how traditional practitioners diagnose their clients. Therefore, it would just involve teaching healers how to identify mental disease and then referring them to biomedical care. They also disagreed with settling offices for traditional healers in health centres	There is a good outlook of integration and openness to work together
Tehrani et al. (2023)	Psychologists did not have sufficient awareness about CAM, and the mean score for their attitude is 40.45 (moderate)	The development and implementation of suitable CAM modality training programs for psychologists is crucial
Thomson‐Casey et al. (2024)	Despite the small numbers of psychologists who believe complementary medicine is not safe for people and recommending it puts people at risk, more than half of psychologists are recommending complementary or traditional healing, and one fourth are referring to complementary medicine when they perceive risk‐related CM. But, particularly, clinical psychologists were the least likely to send their patients to any CM practitioner types	The result could inform the development of complementary medicine‐related education and guidelines for psychologists
Van der Watt et al. (2017)	They reported distrust of traditional healing, a feeling of superiority, and the perception that traditional healing may be a treat for their profession. Their views are explained as follows: “They should be removed from the system; we can actually do this if they can give us the evidence base of their intervention, how they work, when they should work, and when they should work”	Further research is needed to explore the true meaning of shared responsibility for the welfare of the patient
van Niekerk et al. (2014)	There is mistrust among health care providers towards traditional healing, and most of the health care providers reported that traditional healers have no role in mental health treatment. Medical professionals are unaware of the advantages and significance of THPs in delivery health care services	Further research is recommended regarding traditional healing practices, as well integration between occupational traditional and occupational therapy
van Rensburg et al. (2014)	Participants had diverse views on the importance of religion and spiritual therapies for mental illness. Most of them reported that referring patients to spiritual healers may be challenging, and integrating spiritual healers into the existing multidisciplinary health professionals' team is very difficult	Dialogue should be developed, there may yet wok be done in formalising the link between various spiritual workers and local psychiatrists
Ye et al. (2020)	Leading psychiatrists have positive attitudes, but others have a mildly positive attitude, and even others are hesitant to support TM for mental illness	Future collaboration between traditional and complementary medicine and biomedicine is potentially feasible, but higher‐quality evidence is required, and academic communication is also important

## 4 Discussion

This review was conducted to synthesise the attitudes of health professionals towards traditional healing approaches for mental illness in a global context. Even though independent studies had been undertaken, there was an absence of summarised evidence on the positions of health professionals towards traditional healing. Collaborative work between traditional healers and health professionals is needed to provide holistic treatment and address service shortages, as recognised by the World Health Organisation's (WHO) recommendation that health programs incorporate evidence‐based traditional treatment as a resource (WHO 2021). Therefore, this systematic review was summarised using a total of 36 studies. From these studies, two studies scored low according to specific quality assessment criteria due to a small response rate (Ramakrishnan, Dias, et al. 2014) and an integration gap in a mixed‐method pilot study (van Niekerk et al. 2014). Despite this, the studies' overall quality ratings were moderate, leading to no exclusion of studies due to their quality score. Furthermore, it might not be commonly advisable to exclude studies based on their quality in order to show the range of perspectives that provide insightful information about external validity (Carroll and Booth 2015). Thus, this study identified three major categories in health professionals' attitudes towards traditional healing for mental illness.

### 4.1 Negative Attitudes

The majority of studies mentioned negative attitudinal expression of health professionals towards traditional healing of mental illness. Their position may have a detrimental effect on the WHO's recommendation of evidence‐based traditional treatments to close the treatment gap in low‐and middle‐income nations (WHO 2021). In addition, this may be obstructive to policies that incorporate traditional treatment as a resource for mental illness (Blignault and Kaur 2020) and the mhGAP plan of addressing the need by considering non‐specialist mental health professionals to close the treatment gap of mental illness in resource‐limited countries. This is because collaboration between the two treatment modalities promotes task sharing, mutual respect, and recognition (Sangaleti et al. 2017; Soori et al. 2024), and without health professionals' active involvement, it is difficult to achieve collaborative treatment for mental illness.

The first thing that shaped their belief system was their concern about power imbalances or status disparities, which are cited as primary areas of uncertainty regarding the integration of these two therapy approaches (Keikelame and Swartz 2015; Kpobi and Swartz 2018; Van der Watt et al. 2017). In particular, doctors believe that their sense of superiority over faith and traditional healers makes it impossible for them to interact and prevents them from collaborating with traditional healers (Shields et al. 2016). Modern mental health services are perceived as superior, better supported, and encouraged by the government (Osafo 2016; Soori et al. 2024). Similarly, biomedical care providers acknowledged the influence of superiority complexes and positioned themselves as authoritative, validating the power dynamic influencing collaboration (Akol et al. 2018; Shaw and Middleton 2013; Van der Watt et al. 2017). Consistent with other studies (Madiba 2010; Stub et al. 2018), collaborative work between traditional and modern treatments is believed to be a risky activity, and this view hindered them from referring patients who seek traditional therapy for their illness (van Rensburg et al. 2014). A study conducted on the attitudes of health professionals towards traditional healing for other diseases, like human immunodeficiency virus (HIV), concluded that biomedical practitioners were not ready to collaborate with traditional healers and that they preferred the integration to be limited to one‐way referrals from traditional healers to biomedical practitioners (Madiba 2010). Despite being willing to teach traditional practitioners, they were not interested in learning from them, and they had negative views of traditional treatment (Madiba 2010). Health care providers' notion that traditional healing could provide a threat or hindrance to their profession in the future was another focal point to their negative attitude, and this belief led some healers to think that traditional healers ought to be removed from the system (Van der Watt et al. 2017). They believed that if they recommended patients to traditional healers, the patients wouldn't trust them in the future (Musyimi et al. 2016). The results of a South African study on health workers attitudes towards traditional medicine for HIV complement this finding, demonstrating a strong negative attitude among medical professionals (Puoane et al. 2012).

The effectiveness and safety of treatment provided by healers was another issue mentioned by health care providers, since some of them had never seen someone healed after receiving medicine from traditional healers (Kokota et al. 2022). They fear exacerbating symptoms if they consider traditional healing for their patients (Bitta et al. 2019; Neathery et al. 2020). However, their negative attitude was identified to be related to mistrust of traditional healing and considering their practices as lacking scientific basis, education, and an inability to understand medical knowledge among traditional healers (Lampiao et al. 2019; van Niekerk et al. 2014), but other literature showed that traditional healers can provide an effective intervention to people with the symptoms of mental illness (Nortje et al. 2016). Most of the participants with negative opinions mentioned the difficulty of integrating traditional healing into the existing multidisciplinary mental healthcare team (Pham et al. 2021; Shields et al. 2016; van Rensburg et al. 2014). Before recommending collaboration between two health systems, there should be a clear direction for frontline health professionals (Nortje et al. 2016; Van der Watt et al. 2017). While doing so, educating and training on each other's discipline and clinical approaches may facilitate changing the minds of some health care providers who have negative attitudes towards traditional practices for mental illness (Appiah et al. 2018; Soori et al. 2024).

### 4.2 Mixed Attitudes

In the studies identified in this review, some of the health care providers were found to have ambivalent opinions regarding traditional treatment for mental illness. However, some of the participants had a positive expression, while others had doubts about traditional practices for mental illness (Khosla and Goel 2021; Thomson‐Casey et al. 2024). In addition, some health care providers acknowledged the role of traditional healers, specifically in the effectiveness of some diseases, such as anxiety, but not in the treatment of other disorders, such as psychotic, mood, and personality disorders (Hassan and Omer 2020). Despite some health care providers recognising integration between traditional and biomedicine as a possible way to enhance good understanding and knowledge among staff, others identified doubts about the benefits of working closer (Schierenbeck et al. 2018). This finding was also evidenced by other studies, which showed that nurses lacked the confidence to recommend complementary and traditional treatments to their patients, despite having a favourable attitude towards traditional healing (Gyasi et al. 2017). Some professionals perceive traditional healers' activities as fraudulent practices that disturb community and modern health, but others believe in the presence of genuine traditional healers who are effective (Bartholomew and Gentz 2019).

Although some health care providers had positive expressions for traditional healing, they did not refer patients to healers; they preferred to follow their scientific‐practitioner model, embracing a client‐centred approach (Hamilton and Marietti 2017). These findings were supported by another study in which healthcare providers engaged in using traditional approaches but were reluctant to recommend them (Bahall and Legall 2017). Another study conducted in Burundi showed that health professionals display ambivalent views towards healers, despite occasional cross‐referrals between the two approaches (Falisse et al. 2018). Among health care providers with moderate attitudes towards traditional healing, their doubt was due to different healing approaches because the two systems are isolated, and there is no clear guideline on how to communicate with and refer their patients to each other (Masemola et al. 2023). There are other studies that support the ambivalent attitudes of health professionals towards traditional healing for mental health problems. However, while nurses perceived that complementary medicine may improve patients with cancer, they lacked awareness of it (Admi et al. 2017). Similarly, the findings are consistent with the reports of a study conducted in low‐ and middle‐income nations on the perception of integration between two approaches, which noted that biomedical practitioners recognised the benefits of combining both practices but had differences in their conceptualisations of mental illness causation (Green and Colucci 2020).

### 4.3 Positive Attitudes

In this study, there were some results identified that showed relatively positive attitudes of health care providers towards traditional healing approaches for mental illness. Particularly, health care providers have positive views on religious healing since they and most of their proponents prayed and recited biblical texts with patients (Patterson 2023), and their strong engagement led them to organise some workshops with healers. The finding is supported by a study conducted on the attitudes of health care practitioners towards the integration of modern and traditional medicine, in which more than half of biomedical practitioners agreed on the affordability and accessibility of traditional medicine to the users (Tolera et al. 2011), and another study showed that all health professionals believe in the therapeutic effects of traditional and complementary medicine and all have personal experience using it (Abuduli et al. 2016). Some of them have experienced using traditional medicine for themselves (Liem and Newcombe 2019), and in other studies, biomedical and traditional healthcare systems coexist and are used simultaneously with their healthcare (Grant et al. 2013). Health care providers believe traditional healing approaches are important means of managing symptoms and giving support (Ho et al. 2016). In addition, health professionals with a positive attitude have experienced consulting traditional healers for their anxious and depressed patients regularly (Lawrence et al. 2014; Mohamed‐Kaloo and Laher 2014; Sarman and Uzuntarla 2022). The finding is supported by another study conducted among health care professionals in Iran that showed the majority of respondents had a positive opinion towards alternative and complementary medicine (Jafari et al. 2021). Similarly, the study conducted on chronic disease also showed positive attitudes of health care providers towards traditional and complementary medicine (Fan et al. 2018). Furthermore, a study conducted on diabetes showed positive views of health care providers towards using complementary and alternative medicine (Atwine and Hjelm 2017). Health care professionals who positively expressed traditional healing approaches tried to practice collaborative work and patient referral with the traditional healers, and service users benefited from their integrated work (Fan et al. 2018; Lawrence et al. 2014; Mohamed‐Kaloo and Laher 2014).

## 5 Limitations

Although frequently consulted, senior librarians included five databases for the search strategy; additional findings may be obtained from other sources. In addition, we managed some outputs using the title and abstract screening procedures; nonetheless, this screening may have missed some investigations. Moreover, significant information might be overlooked because publications not in English and conducted before 2014, conference reports, dissertations, letters, and brief communications were excluded. Finally, despite our attempts to address globally conducted studies, nearly half of them were from African countries, so the findings may not represent real global features.

## 6 Clinical Implications

Despite the fact that integration is essential to closing the mental illness treatment gap around the world, most of the studies included in this review showed health professionals' negative and mixed attitudes towards traditional healing practices. Therefore, this systematic review is important for policymakers to address major issues like traditional treatment effectiveness, patient safety, and referral systems that influenced health professionals' attitudes towards traditional treatment approaches for mental illness. In addition, the study is evidence for clinicians to establish specific guidelines for mental health nurses on how to communicate and link patients with traditional healers in order to improve mental health service. Likewise, evidence is suitable for mental health nursing educators to develop a curriculum that addresses various cultural groups' health beliefs and practices and incorporate evidence‐based traditional healing approaches. Furthermore, health professionals positive attitudes and mutual work with healers can minimise the stigma that hinders mental health nursing care practices (Ramakrishnan, Rane, et al. 2014; Tyerman et al. 2021). Clinicians' positive attitude is also important to consider transcultural nursing diagnosis and interventions for mental health problems. Finally, the finding may have the clinical value of minimising side effects among people with mental illness who mix both treatment approaches without getting advice from the respective service providers (Nikolić et al. 2018; Rodda et al. 2010).

## 7 Conclusion

To provide accessible, affordable, and effective care for mental illness, the WHO recommended the consideration of traditional treatments as a treatment source. To implement this, there needs to be positive communication and understanding between stakeholders. But large numbers of studies in this review showed the health professionals were not supportive of traditional healing approaches for mental illness. They believe that traditional healers may delay modern health care pathways and may cause harm to service users, and they have no trust in the scientific basis, education, or practices of traditional healers. Therefore, showing policy‐supported evidence of the safety, effectiveness, and overall practice of traditional healing might facilitate health professionals better understanding and collaborative work with healers. In addition, creating culturally appropriate guidelines on communication and referral is important.

## Author Contributions

All researchers contributed to this work and approved the final version for publication.

## Ethics Statement

Ethical approval was not required for this review.

## Consent

Since data was not collected from human participants, patient consent was not required for this review.

## Conflicts of Interest

The authors declare no conflicts of interest.

## Data Availability

All data extracted or analysed for study are included in the results part of this systematic review manuscript.
